# Fyn kinase regulates dopaminergic neuronal apoptosis in animal and cell models of high glucose (HG) treatment

**DOI:** 10.1186/s12860-021-00398-y

**Published:** 2021-12-04

**Authors:** Changhong Tan, Xi Liu, Xiaoshuai Zhang, Wuxue Peng, Hui Wang, Wen Zhou, Jin Jiang, Lijuan Mo, Yangmei Chen, Lifen Chen

**Affiliations:** 1 Department of Neurology, The Second Affiliated Hospital of Chongqing Medical University, 74 Linjiang Road, Yuzhong District, Chongqing, 400010 China; 2 Chongqing Medical University, Chongqing, 400010 China

**Keywords:** High glucose, Dopaminergic neuronal apoptosis, Parkinson’s disease, Fyn, mTOR

## Abstract

**Background:**

High glucose (HG) is linked to dopaminergic neuron loss and related Parkinson’s disease (PD), but the mechanism is unclear.

**Results:**

Rats and differentiated SH-SY5Y cells were used to investigate the effect of HG on dopaminergic neuronal apoptotic death. We found that a 40-day HG diet elevated cleaved caspase 3 levels and activated Fyn and mTOR/S6K signaling in the substantia nigra of rats. In vitro, 6 days of HG treatment activated Fyn, enhanced binding between Fyn and mTOR, activated mTOR/S6K signaling, and induced neuronal apoptotic death. The proapoptotic effect of HG was rescued by either the Fyn inhibitor PP1 or the mTOR inhibitor rapamycin. PP1 inhibited mTOR/S6K signaling, but rapamycin was unable to modulate Fyn activation.

**Conclusions:**

HG induces dopaminergic neuronal apoptotic death via the Fyn/mTOR/S6K pathway.

## Background

Diabetes mellitus (DM) is associated with an increased risk for Parkinson’s disease (PD) [1, 2]. Dysregulated blood glucose levels have been identified as a risk marker for more rapid disease progression in PD [3]. However, the mechanism whereby this occurs is unclear. Experimental studies have suggested that neuronal apoptosis activation [4, 5] may play a crucial role in dopaminergic neuronal loss in the substantia nigra [6]. Interestingly, hyperglycemia in DM patients was reported to induce cortical neuronal apoptosis [7]. In vitro, high glucose (HG) can cause human SH-SY5Y cell apoptosis [8], and this cell line is frequently chosen for PD research [9]. Moreover, in a rat model, long-term hyperglycemia induced dopaminergic neurodegeneration and parkinsonian symptomatology [10]. These data indicate that HG may be involved in dopaminergic neuronal apoptosis in PD. However, the mechanism by which HG causes dopaminergic neuronal apoptosis needs further investigation.

Fyn is a nonreceptor tyrosine kinase. Some researchers have reported that Fyn activation promotes apoptosis in cortical and hippocampal neurons [11, 12], and inhibition of Fyn activity inhibits neuronal apoptosis [13]. Moreover, Fyn was reported to induce PD progression in animal models of the disease [14, 15], suggesting a possible role for Fyn in dopaminergic neuronal apoptotic death in PD. Furthermore, Fyn was reported to be involved in glucose metabolism, and Fyn knockout mice exhibit improved glucose tolerance [16]. Therefore, it is plausible that HG-induced dopaminergic neuronal apoptosis is mediated by Fyn activation, but this has yet to be clarified.

Mammalian target of rapamycin (mTOR)/p70 S6 kinase (S6K) is a well-known regulator of various cellular functions, including apoptosis [17]. mTOR was reported to accelerate neuronal death [18] and to be a risk factor for PD [19]. In a mouse model of PD, levels of phosphorylated mTOR (Ser2448) (p-mTOR) in the substantia nigra were increased [20], and pretreatment with the p-mTOR inhibitor rapamycin partially prevented the cell death induced by 6-OHDA [21], exerting a neuroprotective effect in cellular and animal models of PD [22]. Additionally, mTOR/S6K was reported to be activated by HG treatment in differentiated SH-SY5Y cells [23]; however, the mechanism whereby this occurs is unclear. mTOR is also reportedly regulated by Fyn in erythroblasts [24]. Interestingly, Fyn can be activated by HG [25], indicating a possible role for Fyn in HG-activated mTOR. However, whether HG treatment activates mTOR/S6K via Fyn in differentiated SH-SY5Y cells and animal models and leads to neuronal apoptotic death has yet to be clarified. Furthermore, cleaved caspase 3, a marker of cell apoptosis, was reported to be increased by mTOR activation [26].

Thus, we hypothesized that HG treatment activates Fyn, which promotes mTOR phosphorylation by binding and subsequently activating the mTOR/S6K pathway, ultimately inducing dopaminergic neuronal apoptotic death reflected by elevated caspase 3 levels. In this study, Fyn and mTOR/S6K expression and activation levels in a hyperglycemic rat model and HG-treated differentiated SH-SY5Y cells were assessed. Next, we examined whether inhibition of Fyn had a neuroprotective effect against HG-induced dopaminergic neuronal apoptosis via the mTOR/S6K pathway.

## Results

### An HG diet elevates cleaved caspase 3 levels and induces activation of Fyn and mTOR/S6K in the substantia nigra of rats

Rats were divided into 2 groups: control and HG diet. After 40 days, fasting glucose levels (HG: 12.40 ± 6.04 mmol/L vs. control: 4.61 ± 1.29 mmol/L, *n* = 10, *p* = 0.003) and glucose levels 2 h post glucose challenge (HG: 21.89 ± 9.76 mmol/L vs. control: 7.50 ± 1.70 mmol/L, n = 10, *p* = 0.001) were significantly higher in the HG group than in the control group.

**Fig. 1:**
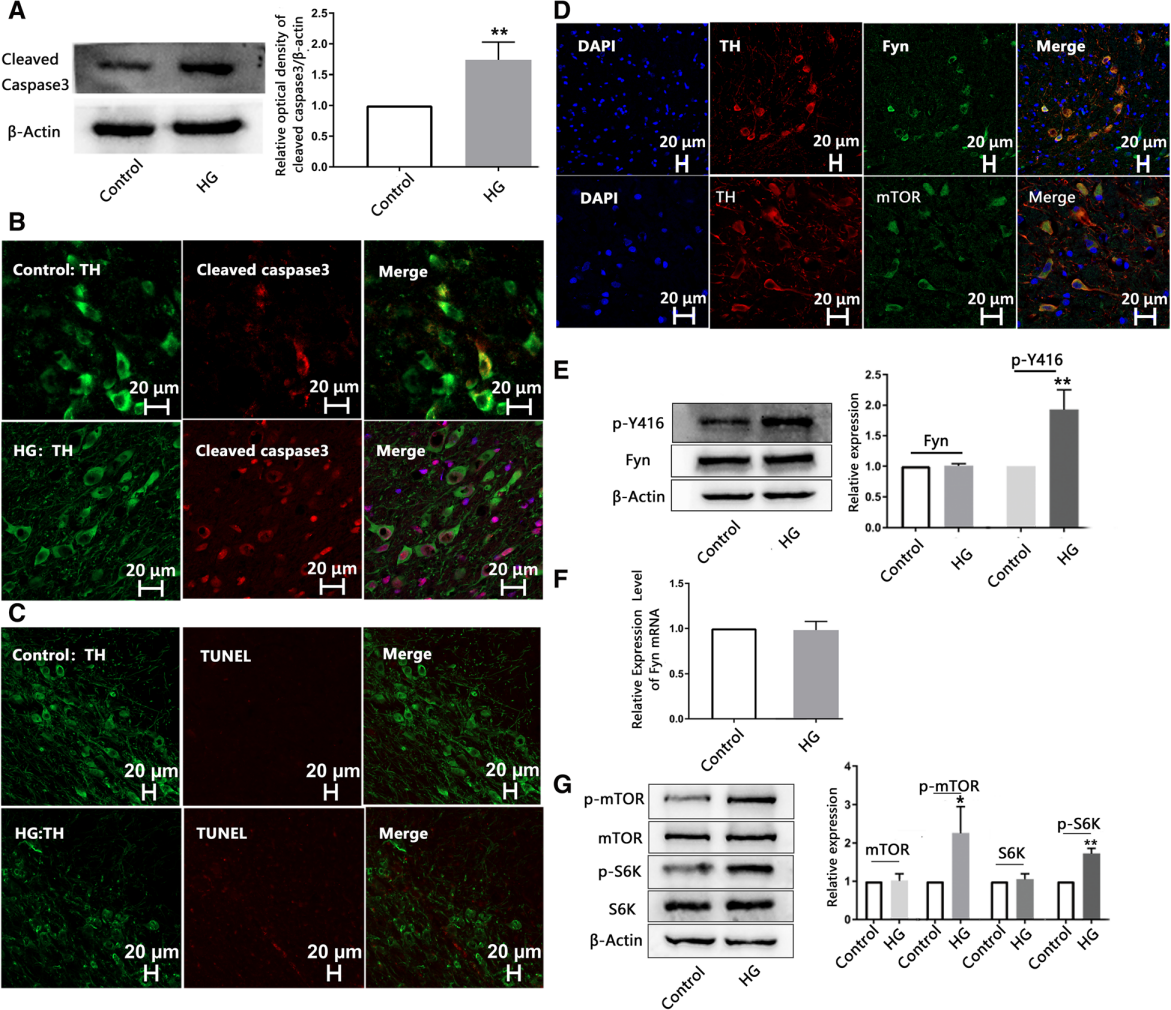
**An HG diet elevates cleaved caspase 3 levels and induces activation of Fyn and mTOR/S6K in the substantia nigra of rats.** Cleaved caspase 3 levels in the substantia nigra of the HG (40 days) group were significantly higher than those in the control group (*n* = 5). The grouping of blots cropped from different parts of the same gel is shown. **(B)** Rat brain sections were stained with cleaved caspase 3 antibody (red) and tyrosine hydroxylase (TH) antibody (green). **(C)** Brain sections were stained with TUNEL (red) and an antibody against TH (green). **(D)** Rat brain sections were stained with antibodies against Fyn or mTOR antibody (red) and TH (green). **(E)** p-Y416 levels in the substantia nigra were elevated in the HG group compared to the control group (n = 5), but Fyn levels were not influenced. The blots of β-actin and Fyn were cropped from different parts of the same gel, and p-Y416 was cropped from different gels. (F) qRT–PCR indicated that mRNA levels of Fyn were unchanged by HG (n = 5). (G) p-mTOR at Ser2448 and p-S6K at Thr389 in the HG group were significantly higher than those in the control group, but total mTOR and S6K levels were comparable (*n* = 5). β-actin and S6K were cropped from different parts of the same gel, and p-S6K, mTOR and p-mTOR were cropped from different gels

The HG diet also increased cleaved caspase 3 levels in the substantia nigra compared to the controls (1.739 ± 0.288 vs. 1, *n* = 5, *p* = 0.005) (Fig. 1A). Immunofluorescence staining for cleaved caspase 3 revealed a positive signal for apoptosis in dopaminergic neurons (Fig. 1B). However, TUNEL staining revealed no obviously positive apoptotic dopaminergic neurons in the HG group (Fig. 1C).

Double labeled fluorescence staining suggested that Fyn and mTOR are localized in dopaminergic neurons in the substantia nigra (Fig. 1D). The HG diet increased levels of phosphorylated Fyn on Y-416 (p-Y416) in the substantia nigra of rats compared to controls (1.928 ± 0.322 vs. 1 *n* = 5,*p* = 0.003). However, total protein levels of Fyn were unaffected (1.008 ± 0.032 vs. 1, *n* = 5, *p* = 0.722) (Fig. 1E). Furthermore mRNA levels of Fyn were also unchanged by the HG diet (0.982 ± 0.096 vs. 1, n = 5, *p* = 0.691) (Fig. 1F). Additionally, the HG diet significantly increased the levels of p-mTOR at Ser2448 (2.259 ± 0.688 vs. 1, n = 5, *p* = 0.015) and p-S6K at Thr389 (1725 ± 0.303 vs. 1, *n* = 5, *p* = 0.006), while total mTOR and S6K levels were unchanged (Fig. 1G). These data indicated that the HG diet activates the Fyn and mTOR/S6K pathways.

### Six-day HG treatment induces apoptotic death and activates Fyn and mTOR/S6K pathways in differentiated SH-SY5Y cells

**Fig. 2:**
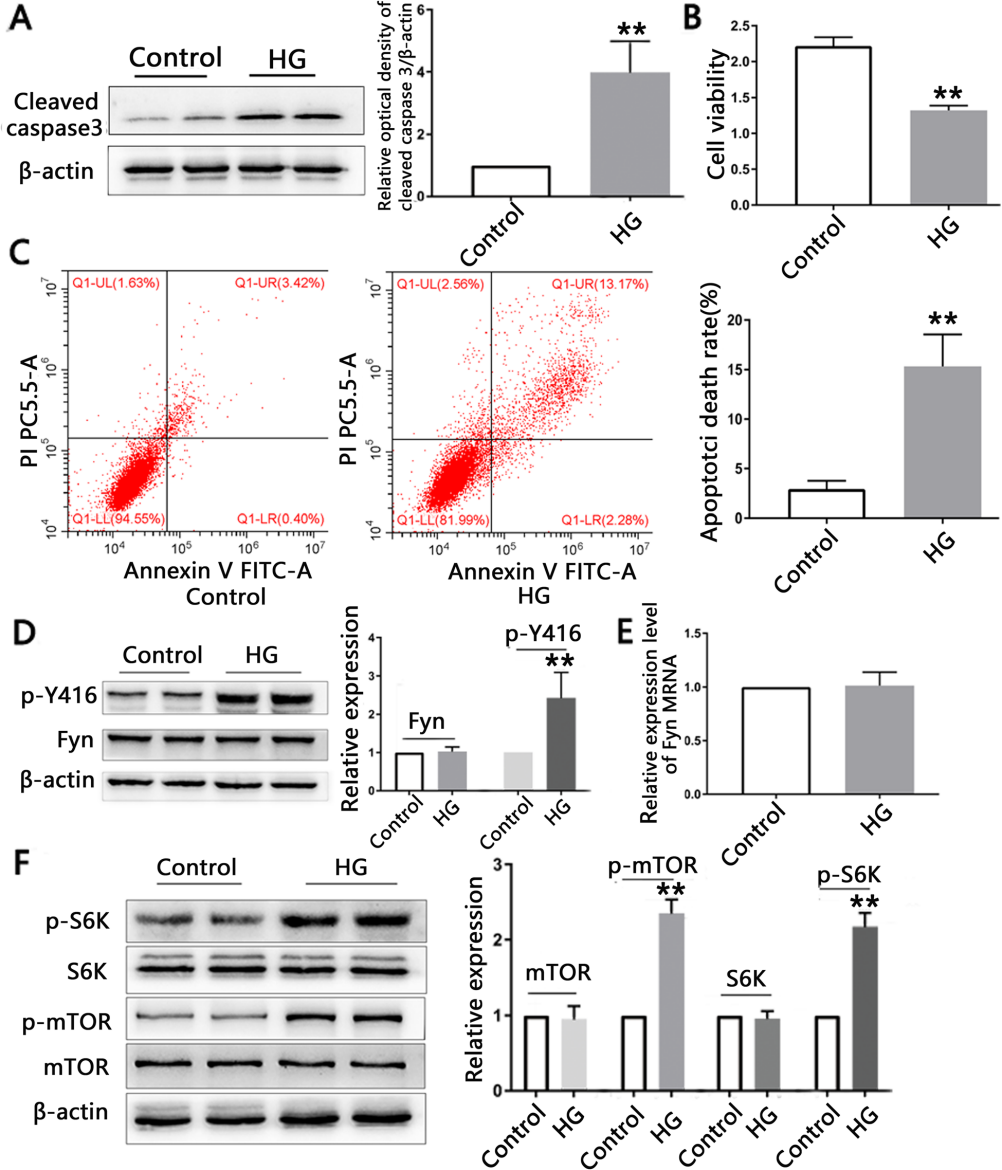
**HG treatment induces apoptotic death and activates Fyn and mTOR/S6K in differentiated SH-SY5Y cells. (A)** Cleaved caspase 3caspase 3 levels in the HG (75 mM, 6 days) group were higher than those in the control group. The grouping of blots cropped from different parts of the same gel is shown. **(B)** Cell viability was significantly lower in the HG group than in the control group (n = 5). **(C)** Annexin V-FITC-positive cells were significantly increased in response to HG treatment compared to controls (*n* = 3). **(D)** p-Y416 was obviously higher in the HG group than in the control group (*n* = 5). The blots of β-actin and Fyn were cropped from different parts of the same gel, and p-Y416 was cropped from different gels. **(E)** mRNA levels of Fyn were not changed by HG treatment (*n* = 3) **(F)** p-mTOR and p-S6K in HG group were significantly higher, but total mTOR and S6K were comparable (*n* = 5). β-actin and S6K were cropped from different parts of the same gel, and the p-S6K, mTOR and p-mTOR were cropped from different gels

Six-day HG treatment significantly increased cleaved caspase 3 levels compared to controls (3.979 ± 1.010 vs. 1, *n* = 5, *p* = 0.003) (Fig. 2A). Cell viability was also significantly decreased in the HG group compared to controls (2.217 ± 0.123 vs. 1.321 ± 0.068, *n* = 5, *p* < 0.001) (Fig. 2B). Annexin V-FITC-positive cells were significantly increased in the HG group compared to controls (15.31 ± 3.24% vs. 2.940 ± 0.819%, *n* = 3, p = 0.003) (Figs. 2C).

HG treatment significantly increased levels of p-Y416 compared to controls (2.426 ± 0.665 vs.1, *n* = 5, *p* = 0.009). However, total protein (1.024 ± 0.120 vs. 1, n = 5, *p* = 0.668) (Fig. 2D) and mRNA levels (1.014 ± 0.125 vs. 1, n = 3, *p* = 0.862) (Fig. 2E) of Fyn were unchanged in response to HG treatment. Additionally, 6-day treatment with HG significantly increased the levels of p-mTOR at Ser2448 (2.349 ± 0.185 vs. 1, n = 5, *p* < 0.001) and p-S6K at Thr389 (2.174 ± 0.181 vs. 1, n = 5, p < 0.001) in the HG group compared to the control group, while total mTOR and S6K levels remained unchanged (Fig. 2F).

### Inhibition of Fyn alleviates HG-induced cell injury by suppressing the mTOR/S6K signaling pathway

**Fig. 3:**
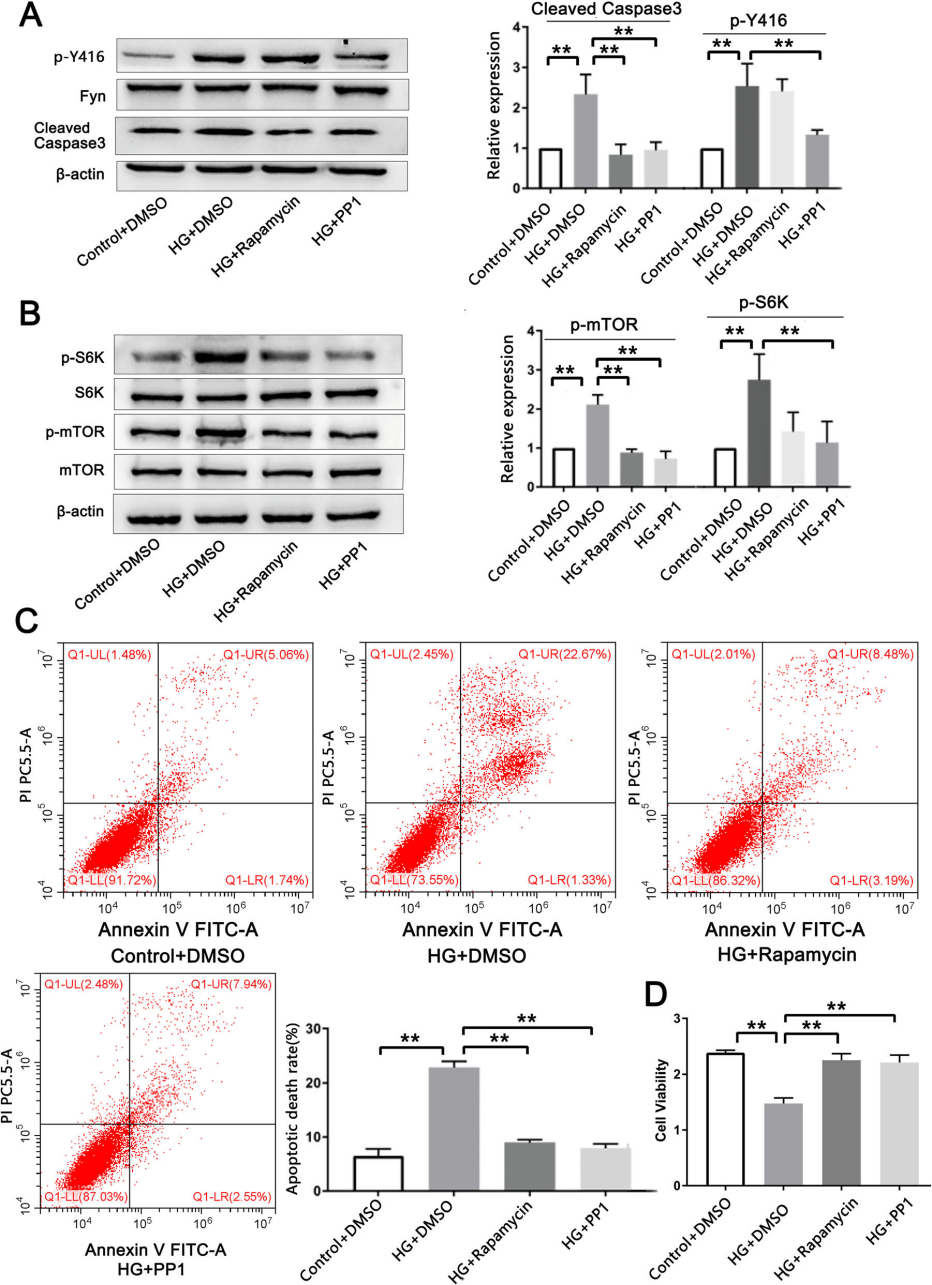
**Inhibition of Fyn alleviates HG-induced cell injury by suppressing the mTOR/S6K signaling pathway. (A)** Elevated cleaved caspase 3 levels induced by HG were rescued by rapamycin (100 nM) or PP1 (10 μM) treatment. PP1 inhibited p-Y416, but rapamycin did not change p-Y416 levels (*n* = 5). β-actin, cleaved caspase 3 and Fyn were cropped from different parts of the same gel, and p-Y416 was cropped from a different gel. **(B)** PP1 or rapamycin inhibited mTOR/S6K (*n* = 5). β-actin and S6K were cropped from different parts of the same gel, and p-S6K, mTOR and p-mTOR were cropped from different gels. **(C)** Flow cytometry showed that rapamycin or PP1 decreased the elevated cell apoptotic death induced by HG (*n* = 3). **(D)** Decreased cell viability in response to HG was rescued by rapamycin or PP1 administration (n = 5)

To investigate whether Fyn regulates HG-induced apoptotic cell death by activating mTOR/S6K, the mTOR inhibitor rapamycin and the Fyn inhibitor PP1 were used. Cells were divided into 4 groups: control+DMSO, HG + DMSO, HG + rapamycin and HG + PP1. Elevated cleaved caspase 3 levels induced by HG treatment were rescued by rapamycin (100 nM) administration [22] (0.833 ± 0.261 vs. 2.339 ± 0.490, *n* = 5, *p* = 0.004) or PP1 treatment (10 μM) [27] (0.952 ± 0.195 vs. 2.339 ± 0.490, n = 5, *p* = 0.008) (Fig. 3A). PP1 reversed the HG-induced elevation of p-Y416 (1.325 ± 0.127 vs. 2.537 ± 0.552, *n* = 5, *p* = 0.03) (Fig. 3A) and simultaneously repressed the activation of mTOR (0.720 ± 0.196 vs. 2.113 ± 0.253, n = 5, *p* < 0.001) and S6K (1.132 ± 0.551 vs. 2.746 ± 0.656, n = 5, *p* = 0.017) induced by HG treatment (Fig. 3B). Rapamycin downregulated the levels of p-mTOR (0.877 ± 0.090 vs. 2.113 ± 0.253, n = 5, *p* = 0.001) and p-S6K (1.420 ± 0.494 vs. 2.746 ± 0.656, n = 5, *p* = 0.039) in the HG + rapamycin group compared to the HG + DMSO group (Fig. 3B); however, rapamycin treatment did not change p-Y416 levels (2.411 ± 0.304 vs. 2.537 ± 0.552, n = 5, *p* = 0.997) (Fig. 3A), suggesting that Fyn acts upstream of the mTOR/S6K signaling pathway.

Furthermore, rapamycin (8.970 ± 0.500% vs. 22.813 ± 1.122%, *n* = 3, p < 0.001) or PP1 (7.913 ± 0.810% vs. 22.813 ± 1.122%, n = 3, p < 0.001) decreased the elevated apoptotic cell death rate induced by HG treatment (Fig. 3C), and cell viability was rescued by rapamycin or PP1 administration (Fig. 3D), suggesting a protective effect of inhibiting either Fyn or mTOR on HG-induced cell injury.

### Fyn may regulate the mTOR/S6K signaling pathway by binding to mTOR

**Fig. 4:**
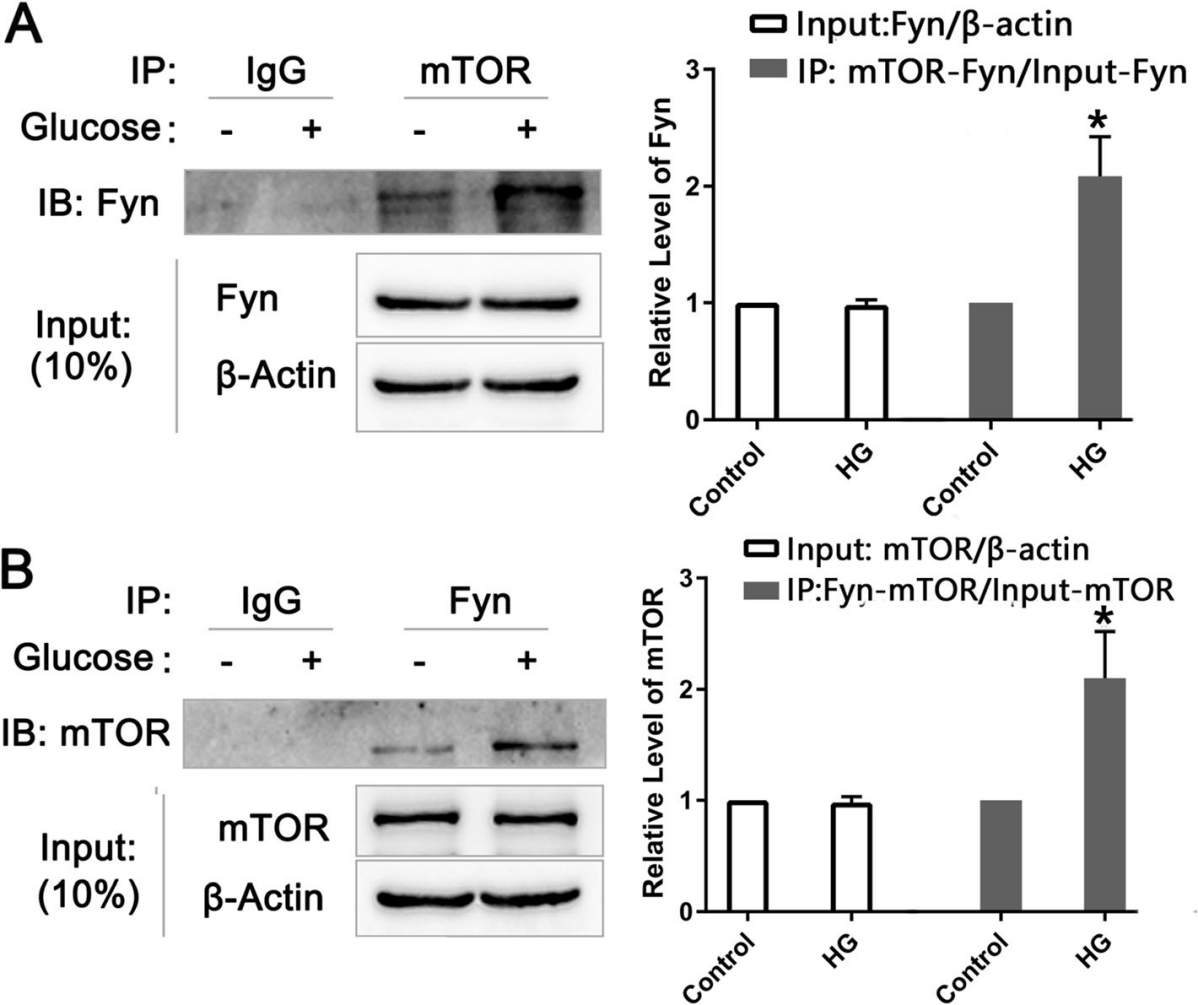
**Fyn may regulate the mTOR/S6K signaling pathway by binding to mTOR. (A)** Quantitative Co-IP analysis indicated that HG treatment elevates mTOR-bound Fyn levels, but Fyn/β-actin input was not changed (*n* = 3). The grouping of blots of input was cropped from different parts of the same gel, and the IB:Fyn was cropped from a different gel. **(B)** Reciprocal experiments validated that Fyn-bound mTOR levels were increased by HG treatment, but the mTOR/β-actin input was not influenced (*n* = 3). The grouping of blots for the input was cropped from different parts of the same gel, and the IB:mTOR was cropped from a different gel

HG-induced apoptosis was mediated by activating the Fyn/mTOR/S6K pathway. However, how Fyn regulates mTOR/S6K signaling is unknown. To address this, we performed quantitative Co-IP. Differentiated SH-SY5Y cells were divided into control and HG groups, and HG treatment elevated mTOR-bound Fyn levels (2.085 ± 0.342 vs. 1, *n* = 3, *p* = 0.005) (Fig. 4A). The reciprocal experiment using an anti-Fyn antibody also validated that Fyn bound to mTOR (2.102 ± 0.420 vs. 1, n = 3, *p* = 0.010) (Fig. 4B) as mTOR levels were increased in response to HG treatment. These data indicated that HG activates Fyn, enhancing binding between Fyn and mTOR and potentially subsequently activating the Fyn/mTOR/S6K signaling pathway.

## Discussion

Activated caspase 3 has been observed in the substantia nigra of PD patients and is described as a vulnerability factor in the apoptotic death of dopaminergic neurons [28, 29]. In our study, cleaved caspase 3 levels were increased in the substantia nigra of rats, and immunofluorescence staining with cleaved caspase 3 revealed a positive signal for apoptosis in dopaminergic neurons. However, TUNEL staining, a late-stage apoptotic assay method, did not identify positive apoptotic dopaminergic neurons in the HG diet group. This may be explained by the relatively short duration of hyperglycemia in this study. Previous data has suggested that in vitro, only long-term hyperglycemia leads to neuronal damage [30], and in vivo, 6 but not 3 months of HG treatment caused dopaminergic neuronal loss in rats [10]. Therefore, 40 days of hyperglycemia exposure may not be long enough to cause neuronal death and positive TUNEL staining. Thus, in vitro, a long-term (6 days) HG treatment procedure was used to explore the effect of HG on neuronal cells in this study.

Mechanisms regarding the link between DM and PD incidence are traditionally speculative; these processes share common cellular mechanisms, such as mitochondrial dysfunction, decreased expression of the transcriptional regulator PPARγ coactivator 1α, and insulin resistance [31, 32]. However, several studies have suggested that hyperglycemia may be an independent pathophysiological risk factor for PD [33, 34]. A prospective cohort study found that DM patients with high fasting plasma glucose were significantly associated with an increased risk for PD (HR:1.23, 95%Cl:1.20–1.25) [33], and another study provided evidence that the risk of PD increases due to impaired fasting glucose (IFG) (HR:1.038, 95%CI:1.009–1.067), a nondiabetic hyperglycemic status, with the authors deducing that the incidence of PD is proportional to the degree or duration of exposure to hyperglycemia rather than simply being dependent on the presence of diabetes [34]. Similarly, we found that a 6-day HG treatment upregulated levels of cleaved caspase 3 and induced apoptotic cell death in differentiated SH-SY5Y cells. These data suggested that the control of hyperglycemia may represent a potential strategy for the management of PD, especially in those with DM. However, in the same study mentioned above [34], patients receiving antidiabetic agents exhibited a high risk for PD. This may be due to an uncontrolled glycemic burden. Moreover, drug use does not imply a substantial intervention in the disease mechanism [34]. Therefore, it is reasonable to speculate that there are other mechanisms involved in the pathogenesis of hyperglycemia leading to PD, and we found that Fyn may represent a new therapeutic target that mediates HG-induced dopaminergic neuronal apoptotic death.

The phosphorylation status of the Y416 residue in the activation loop domain of Fyn was suggested to be a direct indicator of Fyn kinase activation, which can be determined using a p-Y416 antibody [14]. Fyn was reported to be activated by oxidative stress [35], toxic forms of amyloid β protein [36] and cellular prion proteins [37]. However, whether Fyn is activated by HG and subsequently play a role in neuronal apoptosis has not been previously studied. In podocytes, high glucose promotes Fyn activation [25]; unfortunately, the authors did not investigate the mechanism by which HG treatment activated Fyn. As a key molecule regulating energy metabolism, Fyn plays a critical role in cellular responses such as insulin signaling [38], in which glucose metabolism is involved. Otherwise, Fyn is reported to be activated by integrins [39], the expression of which can be elevated by HG treatment [26]. Additionally, HG can cause oxidative injury, which can also activate Fyn [35]. However, whether HG activates Fyn via these mechanisms in differentiated SH-SY5Y cells needs further investigation. In this study, we found that HG treatment activated Fyn, characterized by elevated levels of p-Y416 both in vitro and in vivo, while the proapoptotic effect of HG was abolished by the Fyn inhibitor PP1 in vitro, suggesting that Fyn activity plays a crucial role in HG-induced dopaminergic neuronal apoptotic death and may be involved in dopaminergic neuron loss in PD patients with hyperglycemia. However, we detected no change in total Fyn protein or mRNA levels, which may be explained by Fyn exerting its function primarily by relying on kinase activity [39]. These data indicated that the pro-apoptotic effect of HG is mediated by Fyn activation but not Fyn expression, which was in line with findings in another work, with no alterations in total Fyn expression observed in response to HG treatment [25].

Although the molecular pathway that couples Fyn function to dopaminergic neuronal apoptosis is unclear, mTOR is well known as a regulator of various cellular functions, including apoptosis [17]. Levels of p-mTOR (Ser2448) in the substantia nigra are increased [20], and the mTOR inhibitor rapamycin exerts a neuroprotective effect in cell and animal models of PD [22]. Furthermore, knockdown of S6K also potentiated the inhibitory effect of rapamycin on apoptotic cell death in neuronal cells [40]. These studies indicated that mTOR may activate its downstream effector p70S6K, ultimately leading to neuronal apoptosis. The mTOR/S6K signaling pathway was also reported to be activated by HG [23]. We consistently observed that HG treatment activates Fyn/mTOR/S6K signaling both in vivo and in vitro and that rapamycin suppresses HG-induced neuronal apoptosis in vitro. mTOR is reported to be regulated by several nonreceptor tyrosine kinases, such as Src [41] and spleen tyrosine kinase [42], and it can also be regulated by Fyn [43], which affects glucose metabolism [16]. Thus, we assessed whether activation of mTOR/S6K was induced by Fyn. We found that inhibition of either mTOR or Fyn suppressed HG-induced neuronal apoptosis, and inhibition of Fyn prevented HG-induced activation of mTOR/S6K signaling; however, mTOR inhibition by rapamycin did not suppress Fyn activation, indicating that Fyn acts upstream of mTOR/S6K. These results indicated that HG-induced dopaminergic neuronal apoptosis is mediated by activating the Fyn/mTOR/S6K pathway.

Additionally, how Fyn activates the mTOR/S6K signaling pathway is unclear. We found that HG enhances the binding between Fyn and mTOR through quantitative coimmunoprecipitation, and Fyn activation elevates the phosphorylation levels of mTOR. Similarly, Fyn has been reported to promote the phosphorylation of target proteins, such as the semaphorin receptor, NMDAR, NR2A Tau and α-synuclein, by binding these factors [39]. Furthermore, phosphorylated Fyn activates mTOR in HEK293T cells [44] and skeletal muscle [43], and knockdown of Fyn inhibits phosphorylation of mTOR in cholangiocarcinoma cell lines [45]. These findings provide evidence that activated Fyn phosphorylates mTOR and subsequently activates the Fyn/mTOR/S6K signaling pathway.

The limitation of this study is that we used PP1 to investigate the functional loss of Fyn; however, there may be additional mechanisms by which PP1 may confer neuroprotective effects. Further experiments knocking down or knocking out Fyn gene expression may support the findings that Fyn-mediated HG induces neuronal apoptotic death more strongly. Although the differentiated SH-SY5Y cells used in this study are frequently chosen for PD research, the SH-SY5Y cell line displays a number of genetic aberrations due to its cancerous origin [9], and primary substantia nigra dopaminergic neurons could be a better choice for developing a PD cell model.

## Conclusions

HG treatment activates Fyn, promotes the interaction between Fyn and mTOR, and subsequently activates Fyn/mTOR/S6K signaling, ultimately inducing dopaminergic neuronal apoptotic death. These results revealed a novel mechanism involved in HG-associated dopaminergic neuronal death. Fyn may represent a therapeutic target for preventing HG-related dopaminergic neuronal loss in PD patients.

## Materials and methods

### Animal experiments

Adult male Sprague–Dawley rats weighing 200 to 300 g (Experimental Animal Center of Chongqing Medical University) were housed in a specific-pathogen-free facility with a 12-h light/dark cycle and were allowed free access to standard chow and water for 1 week. Then, rats were randomly divided into 2 groups: control (*n* = 10), which remained on a standard diet until the end of the experiment, and HG diet, which was fed regular chow and had continuous free access to water containing 50% glucose (*n* = 10). Rats were sacrificed by intraperitoneal injection of pentobarbital after a 40-day experimental procedure. Brains were removed for analysis, and their remaining carcasses were transferred to the animal care center.

### Glucose tolerance test

After a 40-day experimental procedure, blood glucose was measured using a glucometer (Roche Diagnostics Scandinavia AB, Bromma, Sweden). Rats were fasted overnight for 16 h. The plasma glucose concentration of tail blood was measured before and 2 h after intragastric injection of 50% glucose (2 g/kg body weight).

### Antibodies and chemicals

Anti-tyrosine hydroxylase (TH) (ab112, Abcam, Cambridge, UK); Anti-TH (sc-25,269, Santa Cruz, Santa Cruz, USA); anti-cleaved Caspase 3 (9664; CST, Danvers, USA); Anti-Fyn (ab184276, Abcam, Cambridge, UK); anti-p-Y416 (6943; CST, Danvers, USA); anti-mTOR (2972; CST, Danvers, USA), anti-p-mTOR (Ser2448) (5536; CST, Danvers, USA); anti-p70 S6 Kinase (9202; CST, Danvers, USA); anti-p-S6K(Thr389) (97,596; CST, Danvers, USA); anti-β-actin (20536–1-AP, Proteintech, Wuhan, China), FITC-conjugated goat anti-rabbit IgG (Proteintech, Wuhan, China); FITC-conjugated donkey anti-mouse (Proteintech, Wuhan, China); Alexa Fluor 555-conjugated donkey anti-rabbit (Beyotime, Haimen, China); PP1 (HY-13084,MCE,New Jersey, USA); rapamycin (HY-10219, MCE, New Jersey, USA); all-trans retinoic acid (RA) (R2625, Sigma–Aldrich, Missouri, USA); glucose (15,023,021,Thermofisher, Waltham, USA).

### Human SH-SY5Y neuroblastoma cell culture, differentiation and treatment protocol

SH-SY5Y cells were purchased from Procell Life Science & Technology Co., Ltd. (Wuhan, China) and cultured in MEM/F12 containing 10% fetal bovine serum (FBS), 25 mM glucose and 1% penicillin-streptomycin (100 units/mL) (Thermo Fisher, Waltham, USA). After plating for 24 h, media containing 10 μM RA was used to induce differentiation on day 1 and was continued for 5 days. On day 4, the medium was replaced with MEM/F12 medium containing 1% FBS and 10 μM RA. On day 6, differentiated cells were maintained in MEM/F12 medium containing 75 mM glucose or 69.5 mM mannitol plus 5.5 mM glucose for another 6 days. All drugs were dissolved in DMSO. Cells were pretreated with PP1 (10 μM) or rapamycin (100 nM) for 1 h before HG (75 mM) treatment and continued with the following procedures. Media was replaced every 2 days.

### Terminal deoxynucleotidyl transferase dUTP nick end labeling (TUNEL) assay

TUNEL staining of brain sections in the substantia nigra from rats was performed using the One Step TUNEL Apoptosis Assay Kit (C1090, Beyotime, Haimen, China). Dopaminergic neurons were labeled with an anti-TH antibody and a related FITC-conjugated secondary antibody. Images were acquired using a laser scanning confocal microscopy (Nikon 1R, Japan).

### Cell viability measurement

MTS analysis was performed using a Celltiter 96 AQueous One Solution Assay Kit (Promega, Madison, USA) to measure cell viability. Briefly, SH-SY5Y cells were seeded into 96-well plates and differentiated with RA for 5 days. After further treatment with drugs (detailed in the cell culture, differentiation and treatment protocol section), cells were incubated with 100 μL of newly added media containing 10 μL of MTS reagent at 37 °C for 4 h. The absorption was subsequently measured at 490 nm using a microplate reader (MultiSkan, GO, Thermo Fisher, Waltham, USA).

### Measurement of apoptotic death

After treatment with drugs, differentiated SH-SY5Y cells were collected and resuspended in phosphate buffer solution. An Annexin V-FITC apoptosis analysis kit (Sungene Biotech, Tianjin, China) was used to identify apoptotic cells. Next, the apoptotic rate was determined using CytoFLEX (Beckman Coulter, California, USA).

### Quantitative real-time polymerase chain reaction (qRT–PCR)

Total RNA from the substantia nigra of brain tissues from rats after a 40-day experimental procedure and drug-treated differentiated SH-SY5Y cells were extracted using RNAiso plus (Takara, Shiga, Japan) and transcribed into complementary DNAs using HiScript 21II Q RT SuperMix for qPCR (+gDNA wiper) (Vazyme, Nanjing, China) on the Applied Biosystems Veriti-Well Thermal Cycler (Thermo Fisher, Waltham, USA). qRT–PCR was performed using ChamQ Universal SYBR qPCR MasterMix (Vazyme, Nanjing, China) on the CFX96 Real-Time System (Thermo Fisher, Waltham, USA). Relative gene expression levels were calculated using the 2^−ΔΔCt^ method [46]. The primer sequences were as follows: Human Fyn: forward: 5′-GGTGTGAACTCTTCGTCTCATA-3′; reverse: 5′-TGTCCGTGCTTCATAGTCATAA-3′. Rat Fyn: forward: 5′-AGCGAAACTGACGGAGGAGAGG-3′; reverse: 5′-GTGCTGAGGGGTGGGGTCTG-3′. Actb2 was used as a housekeeping gene for qRT–PCR in this study.

### Western blot

Total proteins were extracted from substantia nigra brain tissues of rats after a 40-day experimental procedure and differentiated SH-SY5Y cells with respective treatments using RIPA (Beyotime, Haimen, China). Centrifuged protein lysates were mixed with 5× sodium dodecyl sulfate (SDS) loading buffer and boiled for 10 min. Equal amounts of protein were separated by 8–12% SDS–PAGE, gels were cut and then transferred to polyvinylidene fluoride (PVDF) membranes (Merck Millipore, Darmstadt, Germany). Membranes were blocked with QuickBlock Blocking Buffer (Beyotime, Haimen, China) for 30 min at room temperature and then incubated with primary antibodies overnight at 4 °C. β-actin was used as a housekeeping gene. After washing in Tris-buffered saline with Tween-20 (TBST), membranes were incubated with a horseradish peroxidase-conjugated goat anti-rabbit antibody (A21020 Abkine, Wuhan, China) for 1 h at room temperature. Proteins on the PVDF membranes were visualized using a chemiluminescent HRP substrate (WBKLS0100, Merck Millipore, Darmstadt, Germany) and scanned using a Fusion-FX7 image analysis system (Vilber Lourmat, Collégien, France).

### Coimmunoprecipitation

Protein extracts from differentiated SH-SY5Y cells treated with HG or not for 6 days were diluted in cell lysis buffer for immunoprecipitation (Beyotime, Haimen, China) and incubated with 2 μg of rat IgG (Beyotime, Haimen, China), anti-Fyn antibody, or anti-mTOR antibody overnight at 4 °C. Then, Protein A/G agarose (Beyotime, Haimen, China) was added to the mixtures and rotated at 4 °C for 2 h. The mixtures were centrifuged at 1000 g for 5 min, and the precipitates were washed with lysis buffer 5 times. Next, the immunoprecipitates were dissolved in 1× SDS loading buffer and boiled for 10 min. Western blots were performed as described above to verify the interactions between proteins.

### Immunofluorescence staining

Fixed brain tissues were dehydrated in 30% sucrose and sliced into 10-μm-thick frozen sections that were immersed in acetone for 15 min at 4 °C and permeabilized in 0.4% Triton X-100. Then, the sections were blocked with normal donkey serum at room temperature for 1 h after antigen retrieval. Sections were incubated with TH and Fyn, mTOR or cleaved caspase 3 antibody at 4 °C overnight. The sections were then washed with PBS again and incubated with the corresponding secondary fluorescent antibody in darkness at room temperature for 1 h. Then, the sections were stained with DAPI at 37 °C for 10 min and mounted with antifade mounting media. Images were acquired using laser scanning confocal microscopy (Nikon 1R, Japan).

### Statistical analysis

All data were assessed for distribution, and if the data were normally distributed, they are presented as the means±standard deviation (SD). At least three replicated independent experiments were performed. Data analysis and graph creation were performed using SPSS 20.0 software (IBM, Armonk, USA) and GraphPad Prism 6.01 (GraphPad software, La Jolla, USA). The differences between 2 groups were analyzed using Student’s t-test. For comparisons of 3 or more groups, one-way ANOVA with Bonferroni’s or Dunnett’s T3 post hoc analysis was used. *p* < 0.05 was considered statistically significant.

## Data Availability

All data generated or analysed during this study are included in this published article.
